# Meta-analysis of serum and/or plasma D-dimer in the diagnosis of periprosthetic joint infection

**DOI:** 10.1186/s13018-020-01808-1

**Published:** 2020-08-06

**Authors:** Cheng Li, Donara Margaryan, Cristina Ojeda-Thies, Carsten Perka, Andrej Trampuz

**Affiliations:** 1 Charité—Universitätsmedizin Berlin, Corporate Member of Freie Universität Berlin, Humboldt-Universität zu Berlin, and Berlin Institute of Health, Center for Musculoskeletal Surgery (CMSC), Charitéplatz 1, 10117 Berlin, Germany; 2 Hospital Universitario 12 de Octubre, Madrid, Spain

**Keywords:** Periprosthetic joint infection, Diagnostic test, D-dimer, Serum, Plasma, Meta-analysis

## Abstract

**Background:**

The purpose of this meta-analysis was to evaluate the diagnostic value of D-dimer in detecting periprosthetic joint infection (PJI).

**Methods:**

A systematic search and screening of relevant studies was performed in the databases PubMed, Web of Science, and Embase using the following medical subject headings (MeSH) or keywords: “arthroplasty or joint prosthesis or joint replacement or periprosthetic joint or prosthetic joint”, “infection or infectious or infected”, and “D-dimer or serum D-dimer or plasma D-dimer or fibrin degradation products”. Data were subsequently analysed and processed using Meta-Disc.

**Results:**

Seven studies with 1285 patients were included in this meta-analysis. The pooled sensitivity, specificity, positive likelihood ratio, negative likelihood ratio, and diagnostic odds ratio were 0.75 (95% confidence interval [CI] 0.70–0.79), 0.69 (95% CI 0.66–0.72), 3.01 (95% CI 1.84–4.93), 0.32 (95% CI 0.19–0.53), and 10.20 (95% CI 3.63–28.64), respectively. Subgroup analyses showed that the use of serum D-dimer had better sensitivity and specificity than plasma D-dimer for the diagnosis of PJI.

**Conclusions:**

Serum D-dimer was shown to have a better diagnostic value than plasma D-dimer for the diagnosis of PJI. Further research is required for clarification.

## Introduction

The diagnosis of periprosthetic joint infection (PJI) remains a challenge for clinicians due to the lack of a gold standard method [1]. Precise diagnosis is difficult, and the key to the successful management of PJI. Preoperative diagnoses are a preliminary screening tool of suspected infection cases in the early stages and provide valuable information on further diagnostic procedures or help to rule out infection. In recent years, systemic inflammatory markers and synovial fluid biomarkers have been a popular research topic [2, 3]. Compared with blood tests, synovial fluid tests depend more on the personal surgical experience of the physician. Accordingly, a number of test results were decided by the examiner’s subjective judgement, although the joint aspiration procedure can affect the final result [4–7]. Therefore, blood tests may be convenient, more stable and cost-effective, and easier to popularize for diagnosing PJI. Serum erythrocyte sedimentation rate (ESR) and C-reactive protein (CRP) are the most commonly used and first-line blood examinations in the diagnosis of PJI. Both tests are included in the guidelines of the American Academy of Orthopedic Surgeons (AAOS) and Musculoskeletal Infection Society (MSIS) [8]. However, CRP, ESR, and the combination of both have a high sensitivity and low specificity for the diagnosis of PJI [9]. Moreover, the levels of ESR and CRP were reported to be normal in low-virulence organisms [10]. New complementary or alternative serum biomarkers to CRP or ESR are required to further improve the diagnostic accuracy. D-dimer is routinely measured for excluding suspected venous thromboembolic disease in orthopaedics [11, 12]. Serum D-dimer is a promising biomarker in the diagnosis of PJI, with higher sensitivity and specificity than ESR or CRP [13]. The newly defined criteria of the MSIS also included the elevated serum D-dimer, with more than 850 ng/mL as a minor indicator [14]. Nevertheless, recent studies have been controversial regarding the diagnostic value of D-dimer [15, 16]. Although both serum and plasma D-dimer were used for diagnosing PJI [17, 18], their diagnostic value has not yet been assessed. The purpose of the present meta-analysis was to determine the accuracy of serum and/or plasma D-dimer in diagnosing PJI.

## Methods

### Search strategy

We searched electronic databases, including PubMed, Embase, and Web of Science for articles that were published on the diagnostic use of D-dimer in detecting PJI. The first article was published in 2017. Therefore, the search time was set between 2017 and 2019. The search strategy is presented in Table 1. In addition, the reference lists of the included studies and relevant literature on D-dimer were also searched manually to identify potential studies until no additional articles could be found. Duplicated articles were removed in Endnote version X9 reference manager software (Thomson Reuters, New York City, NY).

**Table 1:** Search strategy and results between 2017 and 2019

Number	Medical subject headings (MeSH) or text keywords	Web of Science/PubMed/EMbase (items found)
#1	Arthroplasty or joint prosthesis or joint replacement or periprosthetic joint or prosthetic joint	14,141/16,517/18,358
#2	Infection or infectious or infected	202,428/206,267/281,637
#3	D-dimer or serum D-dimer or plasma D-dimer or fibrin degradation products	1519/1764/3454
#4	#1 AND #2 AND #3	19/20/18

### Eligibility criteria

Articles were selected according to the following inclusion criteria: (1) the diagnosis of PJI was confirmed by the International Consensus Meeting (ICM), Infectious Diseases Society of America (IDSA), MSIS, or European Bone and Joint Infection Society (EBJIS) criteria or any other definition, including clinical signs of infection, presence of sinus tract or purulence around the prosthesis, histopathological examination of periprosthetic tissue indicating acute inflammation or positive culture result from synovial fluid, and periprosthetic tissue samples or sonication fluid [1, 8, 19–21]; (2) the number of true-positive (TP), true-negative (TN), false-positive (FP), and false-negative (FN) values were clearly described or could be calculated by their corresponding sensitivity and specificity in each study; and (3) publications written in English.

### Quality assessment

Search results were screened independently in accordance with the inclusion criteria by two investigators. All included studies were assessed according to the QUADAS-2 guidelines [22]. A third author adjudicated any disagreement in the evaluation of the studies.

### Data extraction

Data extraction was completed independently by two investigators and subsequently rechecked by other investigators involved in the study. The following information was abstracted from the articles: first author, year of publication, country, enrolment period, study design, number of total cases and infected cases, type of prosthetic joint, acquisition time, cut-off, diagnostic criteria, potentially influencing elements, the use of antibiotics, and the sensitivity and specificity of the D-dimer test.

### Statistical analysis

For the analysis of the diagnostic value of the D-dimer test, all statistical analyses were performed using Meta-Disc software (version 1.4, Unit of Clinical Biostatistics team, Madrid, Spain) [23]. The pooled sensitivity, specificity, positive likelihood ratio (PLR), negative likelihood ratio (NLR), and diagnostic odds ratio (DOR) were calculated by a bivariate random-effects regression model. The *I*^2^ statistic was used to assess the heterogeneity of the included studies, with a range of values from 0 to 100%. Significant heterogeneity exists when the *I*^2^ value is greater than 50%, and a value of 0% indicates no observed heterogeneity. If heterogeneity existed, subgroup analysis and meta-regression were performed to explain the potential source of heterogeneity, including the study design, type of blood sample, and cut-offs.

## Results

### Literature search results

**Fig. 1:**
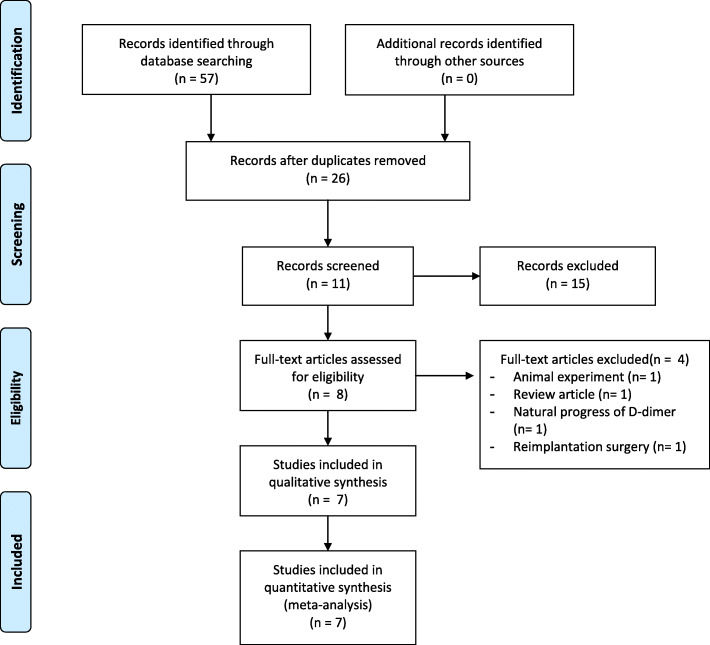
Flow diagram of the selection process for eligible studies

From the selected databases, a total of 57 articles were obtained. Thirty-one articles were excluded due to multiple indexing in different databases. After reviewing the abstracts and full articles, seven articles met the inclusion criteria [13, 15–18, 24, 25]. A flow diagram of the selection process is shown in Fig. 1.

### Characteristics of the eligible studies and quality of the included studies

A total of 1285 hip and knee cases were included in the meta-analysis. The first study was published in 2017 in the USA, and the remaining six papers originated from China in 2019. Among these seven studies, three were conducted retrospectively, whereas the remaining four were prospective studies. Only two studies provided details on antibiotic use. Characteristics of the included studies are summarized in Tables 2 and 3. The QUADAS-2 assessments for each study are shown in Table 4. Results indicated that the included studies were of good quality.

**Table 2:** Characteristics of the included studies for meta-analysis

Study	Country	Enrolment period	Total cases	Infected cases	Study design	Location	Cut-off (ng/mL)	Sen	Spe	Standard	Received antibiotics	Exclusion criteria	Inclusion criteria	Acquisition time
[13]	USA	From April 2015 to August 2016	195	57	Prospective study	Hip, knee	850	89.47%	92.75%	ICM criteria (2014)	Yes	1, 9, 10, 11, 13	2, 15	The day of surgery
[17]	China	From October 2016 to October 2017	30	15	Prospective study	Hip, knee	850	66.67%	60.00%	MSIS criteria (2011)	NA	3, 4, 12, 15		Before surgery
[15]	China	From June 2016 to December 2018	101	31	Retrospective study	Hip, knee	850	71%	80%	ICM criteria (2014)	NA	1, 2, 4, 9, 10, 11, 13, 15		Before surgery
[25]	China	From January 2016 to December 2017	439	76	Retrospective study (multicentre)	Hip, knee	1250	64.5%	65%	ICM criteria (2014)	NA	2, 4, 5, 6, 15		The day of admission
[18]	China	From April 2017 to August 2018	80	26	Prospective study	Hip, knee	756	80.77%	79.63%	MSIS criteria (2011)	NA	1, 5, 9, 11, 15		The day before surgery
[24]	China	From January 2013 to December 2018	318	129	Retrospective Study	Hip, knee	1020	68.29%	50.70%	ICM criteria (2014)	Yes	8, 9, 14	2, 4, 5, 7, 15	The day of admission
[16]	China	From January 2015 to December 2018	122	55	Prospective study	Hip, knee	1170	92.73%	74.63%	ICM criteria (2014)	NA	3, 4, 5, 7, 11, 13, 15		The day before surgery

*Sen* sensitivity, *Spe* specificity, *ICM* International Consensus Meeting, *MSIS* Musculoskeletal Infection Society, *NA* Not available

**Table 3:** Potential influencing elements on D-dimer results

Number	Inclusion or exclusion criteria
**1**	Hematoma
**2**	Systemic inflammatory diseases, such as systemic lupus erythematosus, psoriasis, polymyalgia rheumatica, sarcoidosis, inflammatory bowel disease, gout, hepatitis B and C, lymphocytic leukaemia, myelodysplastic syndrome, and multiple myeloma
**3**	Obesity (body mass index [BMI] > 30 kg/m2), heavy smoking
**4**	Malignancies
**5**	Venous thrombosis
**6**	Cardiovascular and cerebrovascular diseases
**7**	Infection in other regions of the body
**8**	Reimplantation surgery
**9**	History of recent trauma or dislocation (within 2 weeks)
**10**	Any type of skin ulcer
**11**	Visible ecchymosis or a history of hypercoagulation disorder
**12**	Viral infections
**13**	Prosthetic heart valve
**14**	Periprosthetic fracture or joint dislocation
**15**	Inflammatory arthritis

**Table 4:** QUADAS-2 evaluation results

Study	Risk of bias				Applicability concerns		
	Patient selection	Index test	Reference standard	Flow and timing	Patient selection	Index test	Reference standard
Study 1	☺	☺	?	☺	☺	☹	?
Study 2	☺	☺	☺	☺	☺	☺	☺
Study 3	☺	☺	☺	☺	☺	☺	☺
Study 4	☺	?	☺	☺	☺	☺	☺
Study 5	☺	?	☺	☺	☺	☺	☺
Study 6	☺	?	☺	☺	☺	☹	☺
Study 7	☺	?	☺	☺	☺	☺	☺

☺, low risk; ☹, high risk; ?, unclear risk

### Diagnostic value of the D-dimer test for PJI

**Fig. 2:**
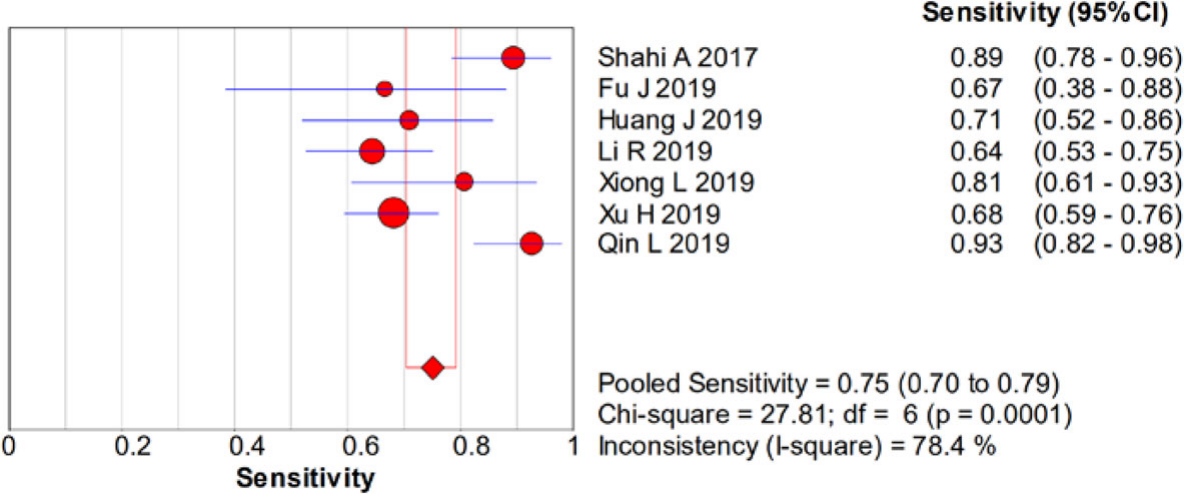
Forest plots of sensitivity of D-dimer for PJI diagnosis

**Fig. 3:**
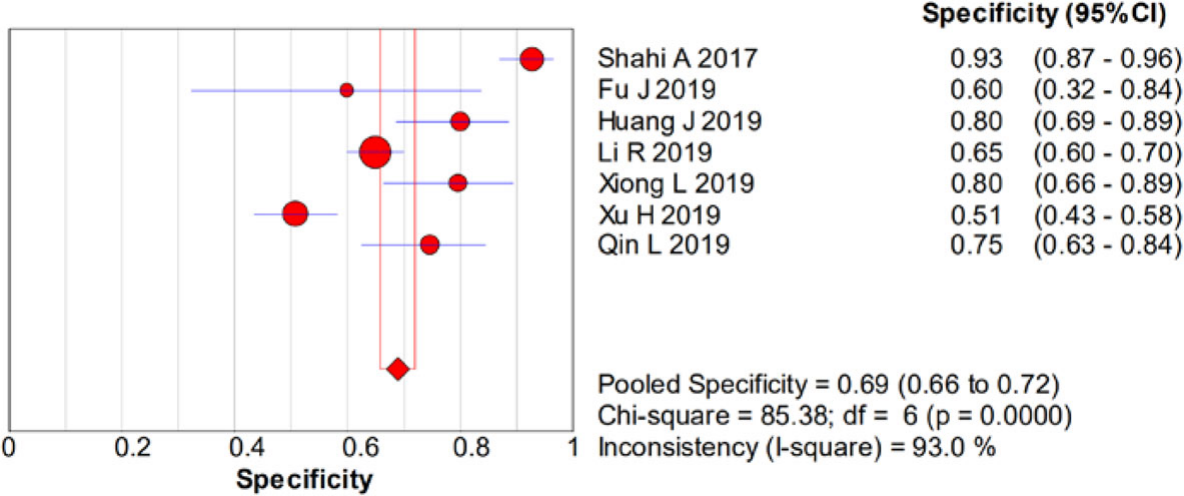
Forest plots of specificity of D-dimer for PJI diagnosis

**Fig. 4:**
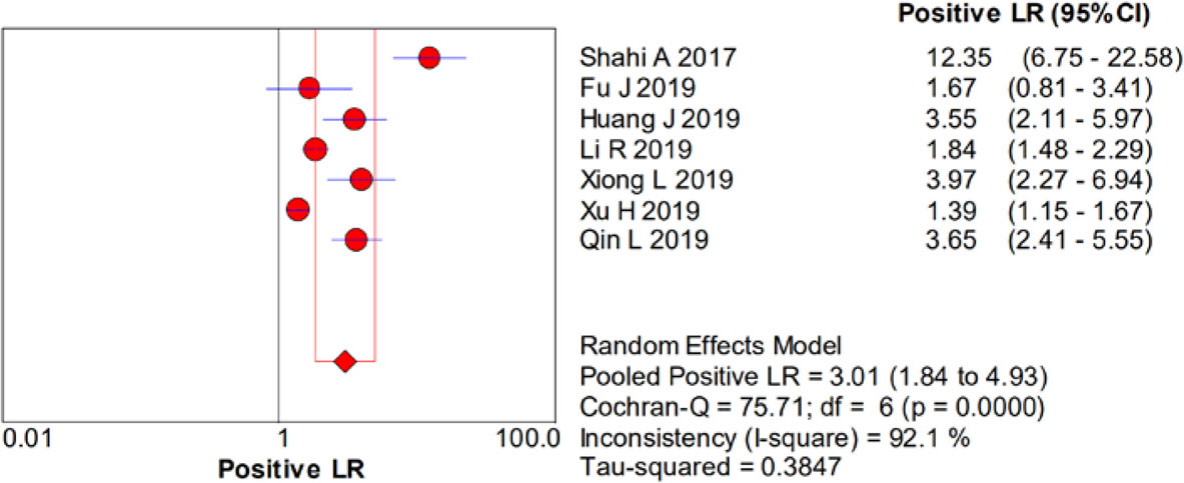
Forest plots of positive likelihood ratio of D-dimer for PJI diagnosis

**Fig. 5:**
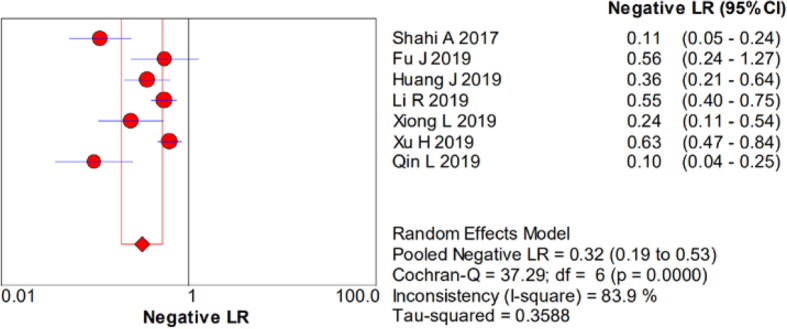
Forest plots of negative likelihood ratio of D-dimer for PJI diagnosis

**Fig. 6:**
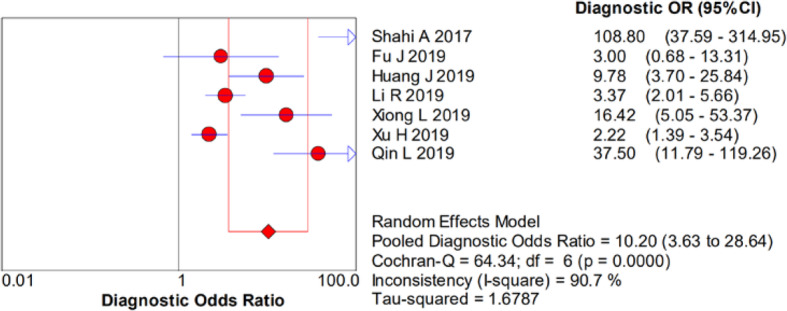
Forest plots of diagnostic odds ratio of D-dimer for PJI diagnosis

**Fig. 7:**
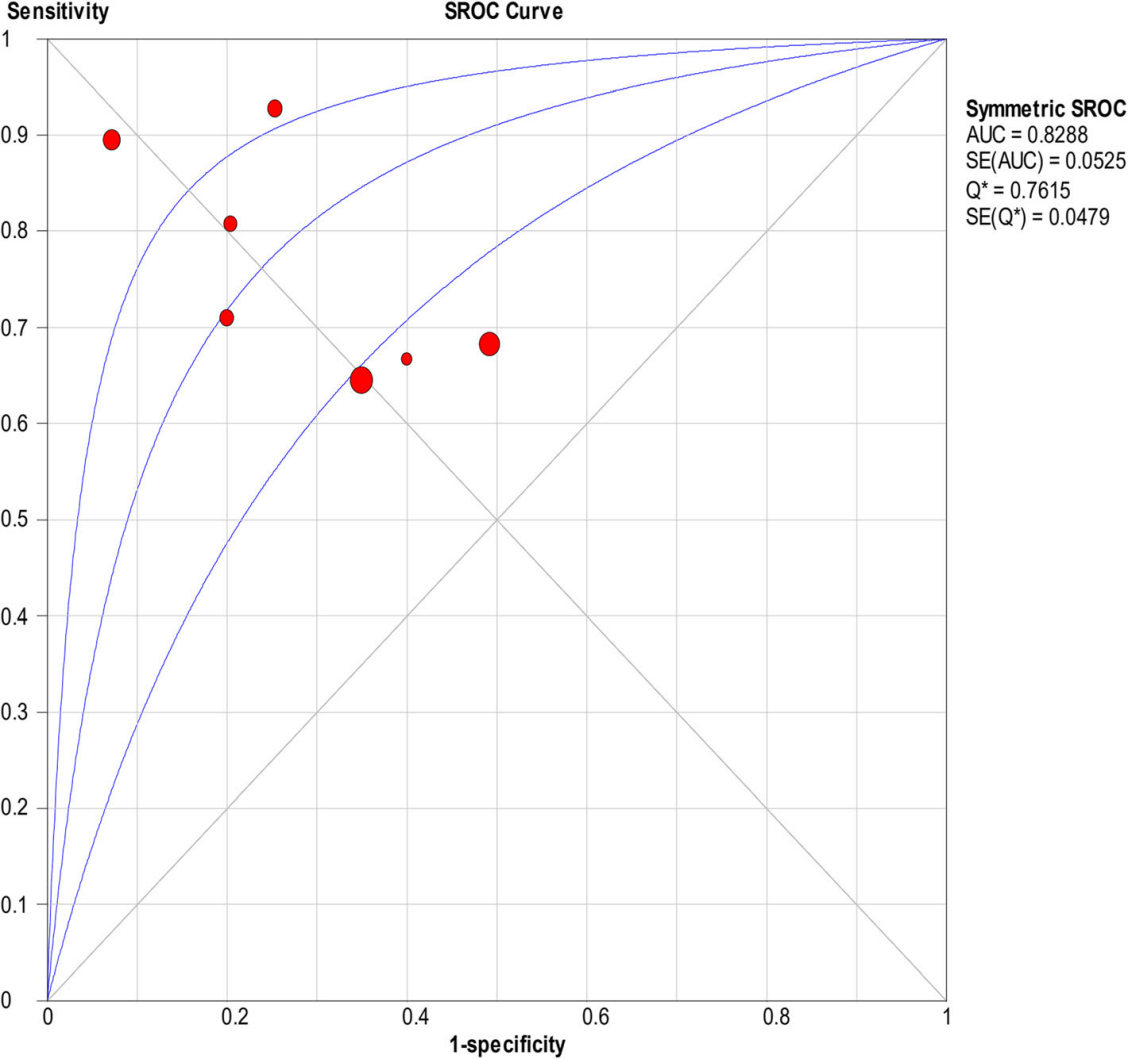
Summary of SROC of D-dimer for PJI diagnosis

There was significant heterogeneity in the sensitivity (*I*^2^ = 78.4%), specificity (*I*^2^ = 93%), PLR (*I*^2^ = 92.1%), NLR (*I*^2^ = 83.9%), and DOR (*I*^2^ = 90.7%). Therefore, a random-effects model was used. The pooled sensitivity, specificity, PLR, NLR, and DOR estimates for the detection of PJI using the D-dimer test were 0.75 (95% CI 0.70–0.79), 0.69 (95% CI 0.66–0.72), 3.01 (95% CI 1.84–4.93), 0.32 (95% CI 0.19–0.53), and 10.20 (95% CI 3.63–28.64), respectively (Figs. 2, 3, 4, 5, and 6). The summary receiver operating characteristic (SROC) plot showed the sensitivity and specificity as well as the 95% confidence intervals and prediction regions, with an area under the curve (AUC) of 0.8288 (standard error, 0.0525; Fig. 7). The subgroup analysis of serum and plasma D-dimer is shown in Table 5.

**Table 5:** Subgroup analysis results

Subgroup analyses	No. of papers	No. of patients	Sen	Spe	PLR	NLR	DOR	SROC (SE)
Overall studies	7	1285	0.75 (0.70–0.79)	0.69 (0.66–0.72)	3.01 (1.84–4.93)	0.32 (0.19–0.53)	10.2 (3.63–28.64)	0.8288 (0.0525)
Type of blood sample								
Plasma	3	787	0.67 (0.60–0.73)	0.60 (0.56–0.64)	1.59 (1.28–1.99)	0.59 (0.48–0.72)	2.69 (1.92–3.77)	0.6599 (0.0261)
Serum	4	498	0.86 (0.80–0.91)	0.84 (0.80–0.88)	4.91 (2.85–8.48)	0.19 (0.09–0.37)	28.25 (9.60–83.15)	0.9096 (0.0332)

*Sen* sensitivity, *Spe* specificity, *PLR* positive likelihood ratio, *NLR* negative likelihood ratio, *DOR* diagnostic odds ratio, *SROC* summary receiver operating characteristic, *SE* standard error

## Discussion

Early diagnosis is the first and most crucial step in the management of PJI. Preoperative testing is at the forefront in assessing an infection, providing valuable information for primary differential diagnosis and further clinical decisions. The measurement of CRP or ESR is a preoperative examination that is a rapid, convenient, simple, and widely used diagnostic method for PJI. However, their diagnostic value is limited due to its low diagnostic accuracy, with levels especially susceptible to fluctuations in patients with dual taper modular stems, slow-growing organisms, and antibiotic treatment [26–28]. Unfortunately, at present, only CRP and ESR appear to be more suitable for the diagnosis of PJI than other serological tests [29]. Shahi et al. [13] first used serum D-dimer (850 ng/mL) for the diagnosis of PJI, showing a higher sensitivity and specificity than ESR and CRP (sensitivity: 89% *vs.* 73% and 79%; specificity: 93% *vs.* 78% and 80%, respectively), even when evaluating the combined sensitivity and specificity of ESR and CRP. In an earlier animal study conducted by Ribera et al. [30], the synovial D-dimer concentration was found to be significantly increased in foals with septic joints (*p* < 0.001). In a prospective study measuring the ESR, CRP, and D-dimer levels before and after primary total hip or knee arthroplasty, the most significant changes in D-dimer levels were observed during the early postoperative period. Levels were sharply increased and peaked on the first day after joint replacement surgery, decreasing to baseline levels on the following day. The author speculated the combination of D-dimer with ESR and CRP might be effective in the early detection of PJI [31]. In the last two years, serum D-dimer was recommended as a promising biomarker in diagnosing PJI and was included in the 2018 ICM criteria for PJI [32].

In the present meta-analysis, the pooled sensitivity and specificity of serum and plasma D-dimer were 0.75 (95% CI 0.70–0.79) and 0.69 (95% CI 0.66–0.72), respectively. The overall diagnostic value of D-dimer had an acceptable sensitivity, whereas the specificity was low. In the subgroup analysis, we found the serum D-dimer to have a better sensitivity and specificity than plasma D-dimer (0.86 and 0.84 vs. 0.67 and 0.60, respectively).

In a prospective study on revision hip and knee arthroplasty, the serum D-dimer had 92.73% sensitivity and 74.63% specificity with a threshold value of 1170 ng/mL [16]. The diagnostic sensitivity and specificity of serum D-dimer were higher than those of CRP (81% and 66%, respectively) and ESR (64% and 70%, respectively), in addition to their combination (89% and 57%, respectively). These results are similar to those of the previously published results of Shahi et al. [13]. However, both studies used different threshold values and differed in the inclusion of systemic inflammatory diseases. In addition, Qin et al. [16] also demonstrated that the combination of serum D-dimer and CRP could achieve the highest sensitivity compared with each alone. However, the sensitivity and specificity of serum D-dimer, CRP, and ESR were reported not to significantly differ when using a serum CRP level of 10 mg/L (68% and 93%, respectively), ESR level of 30 mm/h (74% and 87%, respectively), and D-dimer level of 850 ng/mL (71% and 80%, respectively) as the threshold [15]. In the study performed by Xiong et al. [18], the diagnostic value of serum D-dimer, CRP, and ESR were observed to be equivalent, with results demonstrating the AUCs to be 0.890, 0.831, and 0.838, respectively [18]. From the studies described above, serum D-dimer had a better or equal diagnostic accuracy to CRP and ESR.

In recent years, the diagnostic accuracy of plasma D-dimer for the diagnosis of PJI was also tested. A retrospective cohort study measured the CRP, ESR, interleukin-6 (IL-6), plasma fibrin degradation product (FDP), and D-dimer in diagnosing PJI [24]. The potentially influencing elements included inflammatory disease and antibiotic use. Compared with traditional inflammatory markers, plasma FDP and D-dimer had a lower sensitivity and specificity than CRP, ESR, and IL-6. The sensitivity of the combination of D-dimer and one of the inflammatory marker were decreased compared with the sole use of the indicators, as well as plasma FDP. However, the sensitivity of the combination of D-dimer or one of the inflammatory markers were elevated compared with the sole use of the indicators, and plasma FDP also had similar results. The authors concluded that the diagnostic value of plasma FDP and D-dimer was limited compared to traditional inflammatory markers [24]. A prospective study reported the sensitivity of plasma D-dimer to be between that of CRP and ESR (66.67% vs. 80.00% and 33.33%, respectively), while its specificity was lowest among all three markers (60.00%) [17]. Li et al. [25] showed the diagnostic value of plasma D-dimer to be potentially limited, and the AUC of plasma D-dimer to be inferior to that of plasma fibrinogen, ESR, and CRP (0.657 vs. 0.852, 0.810, and 0.808, respectively). D-dimer only exhibited better performance than that of leukocytes (0.590). Compared with the D-dimer, the diagnostic level of plasma fibrinogen was closer to that of the traditional inflammatory markers ESR and CRP. Moreover, the author also analysed the diagnostic accuracy of D-dimer and fibrinogen with coagulation-related comorbidities (malignancy, autoimmune disease, cardiovascular disease, and cerebrovascular disease). The diagnostic accuracy of D-dimer ranged from 50 to 57.7%, whereas the diagnostic accuracy of plasma fibrinogen ranged from 52.4 to 92.3%. Plasma fibrinogen had better diagnostic accuracy than D-dimer, especially in patients with malignancy. Xu and colleagues assessed the diagnostic value of plasma D-dimer and fibrinogen before reimplantation in two-stage exchange arthroplasty for periprosthetic hip infection [33]. Plasma D-dimer was observed to have a lower sensitivity and specificity than fibrinogen (83.3% and 41.9% vs. 87.5% and 62.8%, respectively); however, it was inferior to fibrinogen. Nevertheless, compared with previous studies on serum CRP and ESR before reimplantation, plasma D-dimer appears to be a better diagnostic indicator [34, 35]. The first serum D-dimer study performed by Shahi and co-workers also found that the two cases of failed second stage replacement caused by reinfection had increased D-dimer levels before reimplantation surgery, while serum CRP and ESR levels were normal [13].

Compared with previous meta-analyses of PJI diagnoses [36, 37], all included papers in this study used similar gold standards. Yet there were several limitations in the current meta-analysis. First, of the seven included studies, six were from China. Whether differences exist in D-dimer values in the diagnosis of PJI in different countries or races is unclear. However, a study performed in the American community-dwelling elderly indicated that black individuals had significantly higher D-dimer levels than white individuals [38]. Further research on whether D-dimer levels are affected by racial differences in normal or PJI patients is required. Second, among these seven papers, only two studies were found describing details of antibiotic use [13, 24]. In addition, all of these publications used different exclusion criteria, which might impact the diagnostic results. Third, from the meta-analysis results, serum D-dimer was found to have a better sensitivity and specificity than plasma D-dimer (86% and 84% vs. 67% and 60%, respectively). However, due to the limited data available, only three studies utilized plasma D-dimer in PJI. Therefore, further PJI studies are required to compare the diagnostic value of serum D-dimer and plasma D-dimer in the future.

## Conclusion

The overall meta-analysis results of serum and plasma D-dimer levels showed low sensitivity and specificity. However, based on the present literature and our subgroup analysis results, serum D-dimer has a better diagnostic value than plasma D-dimer for the diagnosis of PJI; further research is required to verify the present findings.

## Data Availability

Data was extracted from references [13, 15–18, 24, 25]

## Abbreviations

AAOS: American Academy of Orthopedic Surgeons
AUC: Area under the curve
BMI: Body mass index
CI: Confidence interval
CRP: C-reactive protein
DOR: Diagnostic odds ratio
EBJIS: European Bone and Joint Infection Society
ESR: Erythrocyte sedimentation rate
FN: False negative
FP: False positive
FDP: Fibrin degradation product
IDSA: Infectious Diseases Society of America
ICM: International Consensus Meeting
IL-6: Interleukin-6
MeSH: Medical subject headings
MSIS: Musculoskeletal Infection Society
NLR: Negative likelihood ratio
PJI: Periprosthetic joint infection
PLR: Positive likelihood ratio
SROC: Summary receiver operating characteristic
SE: Standard error
Sen: Sensitivity
Spe: Specificity
TN: True negative
TP: True positive

## References

[1] Li C, Renz N, Trampuz A (2018). Management of periprosthetic joint infection. Hip Pelvis..

[2] Berbari E, Mabry T, Tsaras G, Spangehl M, Erwin PJ, Murad MH (2010). Inflammatory blood laboratory levels as markers of prosthetic joint infection: a systematic review and meta-analysis. J Bone Joint Surg Am..

[3] Lee YS, Koo K-H, Kim HJ, Tian S, Kim T-Y, Maltenfort MG (2017). Synovial fluid biomarkers for the diagnosis of periprosthetic joint infection: a systematic review and meta-analysis. J Bone Joint Surg Am..

[4] Nelson GN, Paxton ES, Narzikul A, Williams G, Lazarus MD, Abboud JA (2015). Leukocyte esterase in the diagnosis of shoulder periprosthetic joint infection. J Shoulder Elbow Surg..

[5] Wiler JL, Costantino TG, Filippone L, Satz W (2010). Comparison of ultrasound-guided and standard landmark techniques for knee arthrocentesis. J Emerg Med..

[6] Sendi P, Müller AM, Berbari E (2018). Are all joints equal? Synovial fluid analysis in periprosthetic joint infection. J Bone Jt Infect..

[7] Li C, Renz N, Trampuz A, Ojeda-Thies C (2020). Twenty common errors in the diagnosis and treatment of periprosthetic joint infection. Int Orthop..

[8] Parvizi J, Zmistowski B, Berbari EF, Bauer TW, Springer BD, Della Valle CJ (2011). New definition for periprosthetic joint infection: from the Workgroup of the Musculoskeletal Infection Society. Clin Orthop Relat Res..

[9] Johnson AJ, Zywiel MG, Stroh A, Marker DR, Mont MA (2011). Serological markers can lead to false negative diagnoses of periprosthetic infections following total knee arthroplasty. Int Orthop..

[10] Pérez-Prieto D, Portillo ME, Puig-Verdié L, Alier A, Martínez S, Sorlí L (2017). C-reactive protein may misdiagnose prosthetic joint infections, particularly chronic and low-grade infections. Int Orthop..

[11] Yoo M-C, Cho Y-J, Ghanem E, Ramteke A, Kim K-I (2009). Deep vein thrombosis after total hip arthroplasty in Korean patients and D-dimer as a screening tool. Arch Orthop Trauma Surg..

[12] Jiang Y, Li J, Liu Y, Li Y-C, Zhang W-G (2015). Risk factors for deep vein thrombosis after orthopedic surgery and the diagnostic value of D-dimer. Ann Vasc Surg..

[13] Shahi A, Kheir MM, Tarabichi M, Hosseinzadeh HRS, Tan TL, Parvizi J (2017). Serum D-dimer test is promising for the diagnosis of periprosthetic joint infection and timing of reimplantation. J Bone Joint Surg Am..

[14] Parvizi J, Tan TL, Goswami K, Higuera C, Della Valle C, Chen AF (2018). The 2018 definition of periprosthetic hip and knee infection: an evidence-based and validated criteria. J Arthroplasty.

[15] Huang J, Zhang Y, Wang Z, Dong Y, Zhao Y, Zheng J (2019). The serum level of D-dimer is not suitable for distinguishing between prosthetic joint infection and aseptic loosening. J Orthop Surg Res..

[16] Qin L, Li F, Gong X, Wang J, Huang W, Hu N (2020). Combined measurement of D-dimer and C-reactive protein levels: highly accurate for diagnosing chronic periprosthetic joint infection. J Arthroplasty..

[17] Fu J, Ni M, Chai W, Li X, Hao L, Chen J (2019). Synovial fluid viscosity test is promising for the diagnosis of periprosthetic joint infection. J Arthroplasty..

[18] Xiong L, Li S, Dai M (2019). Comparison of D-dimer with CRP and ESR for diagnosis of periprosthetic joint infection. J Orthop Surg Res..

[19] Parvizi J, Gehrke T (2014). International consensus group on periprosthetic joint infection. Definition of periprosthetic joint infection. J Arthroplasty.

[20] Osmon DR, Berbari EF, Berendt AR, Lew D, Zimmerli W, Steckelberg JM (2013). Diagnosis and management of prosthetic joint infection: clinical practice guidelines by the Infectious Diseases Society of America. Clin Infect Dis..

[21] Zimmerli W, Trampuz A, Ochsner PE (2004). Prosthetic-joint infections. N Engl J Med..

[22] Whiting PF, Rutjes AWS, Westwood ME, Mallett S, Deeks JJ, Reitsma JB (2011). QUADAS-2: a revised tool for the quality assessment of diagnostic accuracy studies. Ann Intern Med..

[23] Zamora J, Abraira V, Muriel A, Khan K, Coomarasamy A (2006). Meta-DiSc: a software for meta-analysis of test accuracy data. BMC Med Res Methodol..

[24] Xu H, Xie J, Huang Q, Lei Y, Zhang S, Pei F (2019). Plasma fibrin degradation product and D-dimer are of limited value for diagnosing periprosthetic joint infection. J Arthroplasty..

[25] Li R, Shao H-Y, Hao L-B, Yu B-Z, Qu P-F, Zhou Y-X (2019). Plasma fibrinogen exhibits better performance than plasma D-dimer in the diagnosis of periprosthetic joint infection: a multicenter retrospective study. J Bone Joint Surg Am..

[26] Kwon Y-M, Antoci V, Leone WA, Tsai T-Y, Dimitriou D, Liow MHL (2016). Utility of serum inflammatory and synovial fluid counts in the diagnosis of infection in taper corrosion of dual taper modular stems. J Arthroplasty..

[27] Shahi A, Deirmengian C, Higuera C, Chen A, Restrepo C, Zmistowski B (2015). Premature therapeutic antimicrobial treatments can compromise the diagnosis of late periprosthetic joint infection. Clin Orthop Relat Res..

[28] Akgün D, Müller M, Perka C, Winkler T (2018). The serum level of C-reactive protein alone cannot be used for the diagnosis of prosthetic joint infections, especially in those caused by organisms of low virulence. Bone Joint J..

[29] Saleh A, George J, Faour M, Klika AK, Higuera CA (2018). Serum biomarkers in periprosthetic joint infections. Bone Joint Res..

[30] Ribera T, Monreal L, Armengou L, Ríos J, Prades M (2011). Synovial fluid D-dimer concentration in foals with septic joint disease. J Vet Intern Med..

[31] Lee YS, Lee Y-K, Han SB, Nam CH, Parvizi J, Koo K-H (2018). Natural progress of D-dimer following total joint arthroplasty: a baseline for the diagnosis of the early postoperative infection. J Orthop Surg Res..

[32] Shohat N, Bauer T, Buttaro M, Budhiparama N, Cashman J, Della Valle CJ (2019). Hip and knee section, what is the definition of a periprosthetic joint infection (PJI) of the knee and the hip? Can the same criteria be used for both joints?: Proceedings of International Consensus on Orthopedic Infections. J Arthroplasty..

[33] Xu C, Qu P-F, Chai W, Li R, Chen J-Y (2019). Plasma fibrinogen may predict persistent infection before reimplantation in two-stage exchange arthroplasty for periprosthetic hip infection. J Orthop Surg Res..

[34] Ghanem E, Azzam K, Seeley M, Joshi A, Parvizi J (2009). Staged revision for knee arthroplasty infection: what is the role of serologic tests before reimplantation?. Clin Orthop Relat Res..

[35] Lindsay CP, Olcott CW, Del Gaizo DJ (2017). ESR and CRP are useful between stages of 2-stage revision for periprosthetic joint infection. Arthroplast Today..

[36] Li C, Ojeda-Thies C, Trampuz A (2019). Culture of periprosthetic tissue in blood culture bottles for diagnosing periprosthetic joint infection. BMC Musculoskelet Disord..

[37] Li C, Renz N, Thies CO, Trampuz A (2018). Meta-analysis of sonicate fluid in blood culture bottles for diagnosing periprosthetic joint infection. J Bone Jt Infect..

[38] Pieper CF, Rao KM, Currie MS, Harris TB, Cohen HJ (2000). Age, functional status, and racial differences in plasma D-dimer levels in community-dwelling elderly persons. J Gerontol A Biol Sci Med Sci..

